# What a NeuroRights legislation should not look like: the case of the Latin American Parliament

**DOI:** 10.3389/fnins.2024.1514338

**Published:** 2025-01-03

**Authors:** Diego Borbón

**Affiliations:** Center for Studies on Genetics and Law, Research Group on Biological Sciences and Law, Universidad Externado de Colombia, Bogotá, Colombia

**Keywords:** neurolaw, neuroethics, neuroprotection, Parlatino, Model Law, regulation

## 1 Introduction

The Latin American Parliament (Parlatino) recently introduced a NeuroRights Model Law to provide a framework for member states to regulate neuroscience and neurotechnologies. However, this initiative suffers from significant theoretical, conceptual, and scientific problems, which raise serious concerns about its application and convenience. This opinion critically examines the key provisions of the proposed law, highlighting its ambiguities, lack of technical rigor, and overreliance on vague ethical-legal concepts. It argues that adopting such a framework could lead to rushed and poorly grounded public policies and neuroscience regulations that fail to address the real challenges posed by neurotechnologies. The article suggests that rather than creating abstract, uninformed, and overly broad regulations, legislators should focus on specific, evidence-based laws, soft-law approaches, international standards, and globally informed principles that address the real risks at the national and international levels. Until then, it is suggested that the Parlatino Model Law should not be incorporated into any legislation and that the proposed law is an example of what a neurorights legislation should not look like.

In 2017, the formal proposal to create a new category of fundamental rights, known as “neuro-rights,” was introduced. This concept was first articulated by Ienca and Andorno (2017), who proposed four core neuro-rights: cognitive liberty, mental privacy, mental integrity, and psychological continuity. In the same year, Yuste et al. published a commentary in *Nature*, further highlighting the ethical issues surrounding neurotechnologies and artificial intelligence (Yuste et al., 2017). As a response, the NeuroRights Initiative, later transformed into the NeuroRights Foundation, was established. This initiative proposed five neuro-rights: mental privacy, personal identity, free will, equitable access to mental augmentation, and protection against algorithmic bias (NeuroRights Foundation, 2024).

Several Latin American countries and regional organizations have advanced reforms to incorporate neurorights into their legal frameworks, employing diverse legislative approaches (Borbón and Ramírez-Gómez, 2024). Chile pioneered these efforts by amending Article 19 of its Constitution to protect psychological integrity and brain activity. After Chile, other countries such as Mexico, Brazil, Argentina, and Colombia are advancing with new bills currently being studied by legislators (Borbón and Ramírez-Gómez, 2024). Soft law approaches have also emerged, such as the Inter-American Juridical Committee's 2023 declaration outlining 10 principles on neurotechnology (Inter-American Juridical Committee – OAS, 2023). Another important regional development is, precisely, the Model Law of the Latin American Parliament.

## 2 The Model Law

The Latin American and Caribbean Parliament, known as Parlatino, is a regional organization composed of the national congresses and legislative assemblies of Latin American and Caribbean states. Its mission is to promote the comprehensive development of the Latin American community, protect fundamental human rights, and combat colonialism and discrimination (Parlatino, 2020).

On May 20, 2023, the Parlatino approved the Model Law on Neurorights which consists of a preamble, 13 articles, and a “General Theoretical and Conceptual Framework Annex.” Its primary goal is to provide a foundation for member countries to legislate and regulate neurotechnologies (Art. 1). It allows flexibility for future advancements, if they align with the philosophical and conceptual bases outlined in the Conceptual Annex (Art. 2), and applies to each country's national territory (Art. 3). The law's main focus is the promotion of neurorights through neuroethics (Art. 4) and outlines a list of new fundamental rights:

- a) Right to mental privacy (the brain data of individuals).
- b) Right to identity and personal autonomy.
- c) Right to free will and self-determination.
- d) Right to equitable access to cognitive enhancement or cognitive development.
- e) Right to protection against biases in algorithms or automated decision-making processes.
- f) The inalienable right not to be subject to any form of intervention in neural connections or any form of brain-level intrusion through the use of neurotechnology, brain-computer interfaces, or any other system or device, without the free, express, and informed consent of the person or user of the device, even in medical circumstances. Even when neurotechnology has the capacity to intervene without the person's awareness.
- g) In general, the right not to be an involuntary or uninformed subject of any process or activity that could interfere with an individual's cognitive processes in any way. This includes practices not directly related to neurotechnology, such as hypnosis and suggestion (Parlatino, 2023, Art. 5, p. 5).

The law further establishes a “Competent Authority” in each country, tasked with 17 specific functions (Arts. 6–7), and outlines mechanisms for universal application, fast-track legal protection for potential violations of neurorights, and broad reparations for harm caused by the state (Arts. 8–10). The final articles call for adaptation of the law to national legal systems, administrative procedures for sanctions, and its eventual enforcement (Arts. 11–13).

## 3 The problematic content of the Model Law

Let's now move on to the several critical observations. First, in the preamble, the law begins by equating the terms “neuro-rights” and “brain rights” (p. 2). As I see it, the “neuro” or “brain” are not adequate concepts as they seem to refer as a protection to the bodily part (brain/neuro), and not to the rights of the person as a whole, which introduces a conceptual error that falls under the mereological fallacy (Borbón et al., 2023). Then, the preamble states that the Model Law is intentionally drafted broadly, and Article 2 further argues that the broad writing is meant to accommodate future advancements so reforms to the law can be “permanently incorporated” (Parlatino, 2023, p. 4). In that sense, proposing a law that requires “permanent” reforms due to its broad scope seems, at least, to be problematic (Borbón et al., 2023).

Articles 4 and 5 then state that the objective of the Law is to promote legislation in member states under the “fundamental criteria” and the “ethical principle of universal validity” of “neuroethics” (p. 4), which might confuse neuroethics as a *criterion* or a *universal principle* rather than the field that studies the “ethical, legal, and social implications of neuroscience and neurotechnology” (Muñoz, 2023). In this regard, it is important to highlight that the concepts used by neurorights initiatives are far from having universal validity or acceptance. See for example the ethical discussion of a neuroright to cognitive enhancement without therapeutic or public health purposes (Herrera-Ferrá et al., 2022; Muñoz and Borbón, 2023) or the conceptual inconveniences of a neuroright to *free will* (Borbón and Borbón, 2021; Muñoz, 2019). As I view it, neuroethics is a research field that might help inform the law, but it is not the role of the law to interpret neuroethics as a universal principle when most neuroethical discussions are constantly moving, changing, and developing through time.

Article 5 then introduces a list of seven new rights, which counted carefully might actually end up being 13 new rights.^1^ But in particular, the list of new rights seems to have worrisome mistakes. For example, list (a) confuses brain data and mental data, which are different (Muñoz et al., 2024). Brain data refers to “quantitative data about human brain structure, activity and function” (Ienca et al., 2022), while mental states and mental data refer to “cognitive, affective, and conative states” such as “thinking, remembering, planning, perceiving, and feeling” (Ienca and Malgieri, 2022). This distinction is crucial because brain data, being quantitatively raw physiological metrics, is more appropriate for applications involving neurological diagnostics or technological interfaces, whereas mental data pertains to those subjective and qualitative aspects inferred by brain data that should be protected by law with more regulatory force. Protecting mental privacy with stronger regulations is essential for safeguarding the important aspects of a person's privacy: emotions, thoughts, feelings, etc. Failing to distinguish between these two concepts might lead to ambiguous or inadequate protections.

Moreover, list (b) contains, in the same concept, the “Right to identity” and “personal autonomy,” which, in our view, are different and should not be mixed as an equal concept. List (c) then contains “free will and self-determination” which may lead one to question exactly what differentiates personal autonomy (list b), from free will (list c), and both of these concepts with self-determination (list c). The Model Law does not clarify the differences or similarities between those new rights. As I see it, identity refers to the attributes, characteristics, and continuity that define an individual as a unique person, encompassing aspects such as personality, social roles, and self-conception, while personal autonomy pertains to the capacity for self-governance and making independent decisions. Although they intersect—since a stable sense of identity often supports autonomous decision-making—they are distinct: identity focuses on “who we are,” whereas autonomy emphasizes “what we decide and how we act.” On the other hand, concepts such as free will and self-determination also have very important philosophical debates that seem to be ignored or not explained by the Model Law, such as the debate on the compatibility of free will and determinism (Kane, 2012).

In list (d), the Parlatino creates a right to “equitable access to cognitive augmentation” which is then confused with “cognitive development” (p. 5), a problem that also resonates in list (e) which confuses “AI bias” with “automatic decision processes” (p. 5). It is worth mentioning that the proposed neuroright to equal access to mental augmentation raises significant ethical concerns, as it risks promoting transhumanist applications that could pressure individuals into enhancements that modify human nature and end up infringing personal freedom (Borbón and Borbón, 2021).

Additionally, such a right may impose an unsustainable financial burden, as it might be understood as a benefit right that must be financed by the states at the expense of “already underfunded health systems” (Muñoz and Borbón, 2023). A right conceived in that way basically means opening the door “to unlimited corporate interests for those companies that develop neurotechnologies, since it would be financing, with public funds, the numerous acquisitions of technologies whose purposes are not therapeutic, nor for public health, in the name of a new ambiguous human right” (Borbón and Borbón, 2021).

The right in list (f) prohibits any intervention in the absence of consciousness and informed written consent, “even in medical circumstances” (p. 5). This, in essence, means an absolute prohibition of medical intervention in any case a patient is unconscious. Take for example a patient who arrives unconscious at the emergency room after a car accident. Under the Parlatino Model law, medical personnel cannot “intervene in the absence of the person's own consciousness” even “under medical circumstances” (p. 5). Consider with the above, the immediate and serious consequences for medicine, clinical research, mental health, and public health policies in the region. As a final note, list (g) prohibits any involuntary intervention including “other practices not necessarily directly related to neurotechnology, such as hypnosis and suggestion” (p. 5), which might lead one to question what hypnosis has to do with the regulation of neurotechnology, or even if a valid psychotherapeutic approach, as hypnosis is (Valentine et al., 2019; Rosendahl et al., 2024), should be prohibited under a neurorights bill.

If the above is not worrying enough, due to the obvious lack of conceptual and technical rigor, let me make a few final comments on the “General Conceptual Theoretical Framework Annex.” The aforementioned Annex states that “The definitions contained in this model law are taken from reliable sources” (p. 2). However, of the more than 90 footnotes that can be extracted from the Annex document, the vast majority of them are Internet sources, including press releases, media interviews, online encyclopedias, including Wikipedia, recordings of the Chilean Senate, lectures by Professor Rafael Yuste, online blogs, and YouTube videos (Borbón et al., 2023). Of the few bibliographical sources that might have some academic content, most are popular books from several decades ago, and others border on science fiction literature. There is not a single high-impact and reliable research paper from the last 5 years on the subject of regulation (Borbón et al., 2023).

Carrying out a practical exercise on August 15, 2023, I uploaded the 18 pages that make up the “General Theoretical Conceptual Framework” Annex to the analysis of “Turnitin” software (An internet-based similarity detection service), finding a high score of coincidences (Borbón et al., 2023). Sometimes, the sources of information are not cited at all, or they cited the wrong sources, or they were not cited based on an adequate referencing system. Specifically, the percentage of coincidences reached 69% (Borbón et al., 2023). The percentage of coincidences does not imply, *per se*, a finding of plagiarism but at least raises important concerns regarding the technical content of the Model Law. If that is not alarming enough, consider that even the last footnote on page 4 of the Conceptual Annex does not even cite the source of information but, after copying and pasting three blog links from internet, cites as the source of information the expression “- otras varias” (p. 4), that is, “other various.” This way of citing the source of information is not in line with any international copyright legislation.

## 4 Conclusions

This opinion briefly examined the Parlatino NeuroRights Model Law and highlighted significant deficiencies in its theoretical, conceptual, and scientific foundation. The vague definitions of neurorights and potential ambiguity in their implementation, combined with a lack of academic rigor, suggest the need for a thorough review. Rather than rushing into abstract and premature new neurorights, I advocate for a more cautious approach that focuses on adapting and reaffirming existing lists of rights, prioritizing soft law and principle-based approaches, and developing precise international and globally informed national regulations to address real neurotechnological risks with multilayered protection. This should be in line with current recommendations from the United Nations Human Rights Council (2024), OHCHR; OECD (2019), Council of Europe (2023), and the Inter-American Juridical Committee – OAS (2023), which, instead of advocating for new lists of rights, make soft law and principle-based recommendations.

As I see it, current human rights frameworks are sufficient and important enough to address challenges from neurotechnology and artificial intelligence, requiring adaptation and reinterpretation, if needed, to protect citizens' rights. In this line, national and regional courts and judges can find useful instruments such as the Inter-American Juridical Committee – OAS (2023) declaration on the Interamerican Principles for Neurosciences and Neurotechnologies to serve as a guide to adjust current human rights interpretations to new challenges. Soft-law instruments, such as those being developed by UNESCO Ad Hoc Expert Group (2024), United Nations Human Rights Council (2024), OECD (2019), Council of Europe (2023), and the Inter-American Juridical Committee – OAS (2023), might serve as guides for judges and legislators. Meanwhile, national and international strong regulations also seem to be necessary for specific areas such as criminal law, regarding protections for privacy, bodily integrity, freedom of thought, and consent, as the punitive power of States might be tempted to use approaches such as non-consensual lie detection, neuroprediction, and coercive neurointerventions (Díaz-Soto and Borbón, 2022). Also, laws regarding commercial access to neurotechnology, or reforms to privacy bills with mental data as personal biological sensitive data might also need to be adjusted with concrete legislations.

All these comments resonate under global academic criticism of neurorights (Borbón and Borbón, 2021; Bublitz, 2022, 2024; Díaz-Soto and Borbón, 2022; Fins, 2022; Fyfe et al., 2022; Ligthart et al., 2023; Moreu Carbonell, 2021; Ruiz et al., 2021). This is a call for a careful, well-informed global debate to ensure that any future legislation is conceptually sound, ethically responsible, and practically applicable. Until then, the Parlatino Model Law is an example of what a neurorights legislation should not look like.

## Footnotes

^1^If I may list: (1) Right to mental privacy; (2) Right to brain data; (3) Right to identity; (4) Right to personal autonomy; (5) Right to free will; (6) Right to self-determination; (7) Right to equitable access to cognitive enhancement; (8) Right to cognitive development; (9) Right to protection against biases in algorithms; (10) Right to protection from automated decision-making processes; (11) Right not to be subject to any form of intervention without consent even in medical circumstances; (12) Right not to be an involuntary or uninformed subject of any process or activity that could interfere with an individual's cognitive processes in any way; (13) Right to not be subject to hypnosis and suggestion.
